# Determinants of impact of a practice accreditation program in primary care: a qualitative study

**DOI:** 10.1186/s12875-015-0294-x

**Published:** 2015-07-03

**Authors:** Elvira Nouwens, Jan van Lieshout, Michel Wensing

**Affiliations:** Scientific Institute for Quality of Healthcare (IQ healthcare), Radboud University Medical Centre, P.O. Box 9101 , 114 IQ healthcare, 6500 HB Nijmegen, The Netherlands

**Keywords:** Practice accreditation program, CVRM, Qualitative study, Primary care, Implementation

## Abstract

**Background:**

Practice accreditation is a widely used method to assess and improve the quality of healthcare services. In the Netherlands, a practice accreditation program was implemented in primary medical care. We aimed to identify determinants of impact of a practice accreditation program, building on the experiences of primary care professionals who had participated in this program.

**Methods:**

An interview study was done to document the experiences of 33 participating primary care professionals and used to identify determinants of outcomes. The Consolidated Framework for Implementation Research (CFIR) was used as framework for the qualitative analysis.

**Results:**

After analyzing 23 interviews saturation was reached. The practice accreditation program is based on structured quality improvement, but only some of its elements were identified as determinants of impact. Factors that were perceived to facilitate implementation of the program were: designating one person responsible for the program, ensuring clear lines of communication within the whole practice team and having affinity with or stimulate enthusiasm for improving quality of care. Contextual factors such as participation in a care group and being connected to the GP educational institute were important for actual change. The accreditation program was perceived to have positive effects on team climate and commitment to quality of care in the practice team. The perception was that patient care was not directly influenced by the accreditation program. Receiving a certificate for completing the accreditation program seemed to have little added value to participants.

**Conclusions:**

Practice accreditation may have positive outcomes on quality of care, but not all planned elements may contribute to its outcomes. Both factors in the accreditation process and in the context were perceived as determinants of quality improvement. The challenge is to build on facilitating factors, while reducing the elements of accreditation that do not contribute to its impact.

**Electronic supplementary material:**

The online version of this article (doi:10.1186/s12875-015-0294-x) contains supplementary material, which is available to authorized users.

## Background

Accreditation and certification are widely used methods to assess and improve healthcare services. These are complex interventions, which typically comprise an audit of a healthcare provider, an assessment of performance, followed by formal allowance of accreditation or certification. Accreditation programmes can have positive effects on quality and safety of clinical care and organizational performance [1]. Worldwide, accreditation focuses on promoting continuous improvements, applying standards and providing feedback [2]. Given the opportunity costs involved, it is important to know which components and contextual factors contribute to the outcomes of accreditation on quality and outcomes of healthcare. However, little is known about this.

In the Netherlands, primary care practice accreditation is a voluntary activity comprising a comprehensive audit, which covers clinical and organizational domains, followed by structured planning of improvements and formal review by an external assessor [3]. The program was initiated by the Dutch College of General Practitioners (DCGP) and is delivered by an independent organization (Netherlands Institute for Accreditation in Healthcare, NPA). While accreditation may serve several purposes, improvement of professional performance and practice organization are prominent among these in the Dutch program [4–6]. Previous research with respect to the Dutch practice accreditation program showed that general practitioners (GPs) were willing to assess their practice in order to improve the quality of care. Furthermore the practice accreditation program is used to obtain understanding of the practice organization in order to enhance the quality of care in the practice [7].

The aim of this study was to identify determinants of impact of the practice accreditation program, building on the experiences of primary care professionals who had participated in this program.

## Methods

### Study design

A qualitative study was conducted, which was linked to a cluster randomized trial of the practice accreditation program in the Netherlands [8]. All participating practices participated in the practice accreditation program and all were invited for the qualitative study. We used semi-structured interviews with participating primary care professionals to identify relevant factors. The Consolidated Framework for Implementation Research (CFIR) was used as framework for analysis [9].

The study was undertaken to identify determinants of impact of the practice accreditation program, building on the experiences of primary care professionals who had participated in accreditation program in primary care; therefore a qualitative method was appropriate.

### Setting

The primary care practice accreditation program in the Netherlands has been offered on a voluntary basis since 2005. Practices have to comply to few minimum standards in order to be eligible for participation [10]. The practice accreditation program comprises, firstly, a comprehensive audit (using validated performance indicators) and written feedback to the practice, which covers a range of clinical domains (cardiovascular risk management (CVRM), diabetes mellitus (DM), asthma and COPD), practice management, and patient experiences. The feedback, which consists of a comparison with benchmarks of other primary care practices, is discussed with a trained observer in a feedback consultation with the whole practice team and helps to identify substandard performance domains. The second obligatory component, the planning of improvements in the practice according to the principles of quality management, are based on this feedback. The practice team may chose to rely on a trained consultant to develop an improvement plan. Participants who perform the procedure as planned are all accredited, so accreditation does not imply that a certain minimum score on performance indicators has been obtained. In the practice accreditation program validated instruments are used: VIP [11], clinical indicators [7] and Europep [12]. Participants receive a certification for the time period of one year which demonstrates (to the public) their involvement in continuous quality improvement. Every year the practice will be audited and every year new improvement plans have to be formulated which have to be approved by the auditor. The prolongation of the accreditation depends on having met the objectives of the improvement plans.

### Participants

Participants in the study were team members of the primary care practice with a coordinating role in the implementation of the practice accreditation program in the primary care practice.

### Interviews

One semi-structured interview with one team member per practice was conducted. All interviews were held by one person (EN), a health scientist and physiotherapist. All 41 practices included in the cluster randomized trial [8] were approached to participate in the interviews. An interview guide was used and was adjusted during the process of interviewing based on interim reviewing of the results. Interviews (by telephone due to feasibility) lasted approximately half an hour.

The interview guides were developed on the basis of literature [7] and during several core-group meetings. Topics that guided the development of the interview were: reasons to participate in the practice accreditation program, consequences of participating in the program, preparation and implementation of improvement plans, incentives for quality improvement, dealing with feedback and the significance of participating in the accreditation program.

### Data analysis

All interviews were audio-taped and transcribed verbatim. Interviews were repeatedly read and analyzed in an iterative approach by three researchers, who independently coded the transcripts of the interviews, followed by collaborative interpretation and consensus. We came to agreement after discussion.

**Fig. 1:**
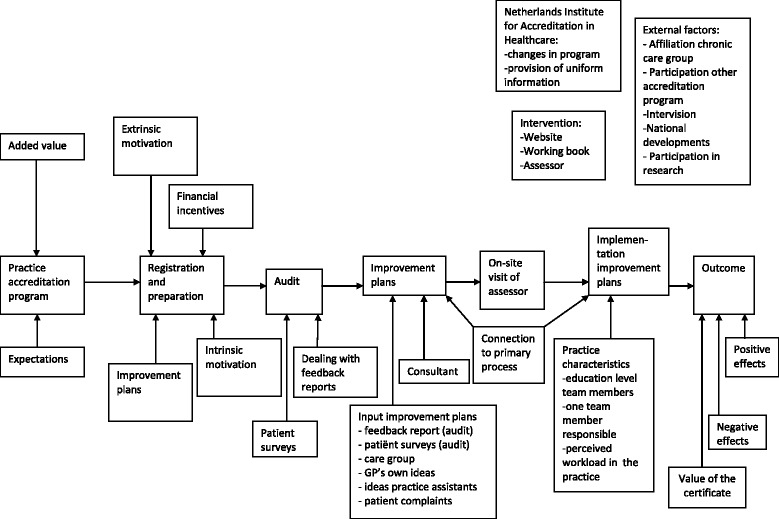
Steps in the practice accreditation program

Interview data were analyzed until saturation was reached, that is, no new codes were generated. A stepwise analytical approach was used [13]. We provisionally coded all statements referring to program components or contextual factors which appeared to influence the impact. In a second stage, the codes were linked to the logical steps in practice accreditation (Fig. 1). We then used the CFIR framework, which provides constructs from multiple domains for identifying potential influences on implementation of interventions [9], in a deductive analysis. The CFIR constructs are organized into five major domains and, as applies to this study, are: characteristics of the practice accreditation program (evidence strength and quality, relative advantage, adaptability, complexity, design quality and packaging, cost); the outer setting (patient needs and resources, cosmopolitanism, peer pressure, external policies and incentives); the inner setting; the process used to implement the program; characteristics of individuals involved (Additional file 1) [9].

### Ethical approval and informed consent

The Medical Ethics committee Arnhem-Nijmegen waived approval for this trial after assessing the study protocol (file number 2008/258). Participants all consented to recording of the interviews. All data was coded and processed anonymously.

## Results

All 41 participating practices were invited for the interview study. Eight practices declined to participate in the study due to lack of time or sickness among staff. Therefore, interviews were held with 33 individuals in the year 2012. Interviews lasted from 17 min until 46 min. Table 1shows characteristics of the interviewed participants. After analyzing data of 23 interviews, saturation was reached. Eight interviews were analyzed and coded by all three authors, 15 interviews were coded by two of the authors (JvL, EN). The linking of codes to the logical steps in the practice accreditation program and to the CFIR framework was performed by all authors. The findings are reported regarding the five domains of CFIR and are supported by verbatim quotations from interviews.

**Table 1:** Characteristics of interview participants

Team member	22 (96 %) GP
	1 (4 %) Practice assistant
Type of practice	10 (44 %) Solo practices
	7 (30 %) Duo practices
	6 (26 %) Group practices
Female	10 (44 %)
GP training practices	20 (87 %)
Participating in care group concerning CVRM	3 (13 %)

### Views on intervention characteristics

#### Intervention source

The practice accreditation program was externally developed by the DCGP [14]. Elements of the program corresponded with existing work processes, which was perceived as beneficial for implementation of the program.

#### Adaptability

The adaptability of an intervention is the degree to which the intervention can be tailored to the needs of the organization. A core component in the practice accreditation program is developing improvement plans on four chronic conditions mostly treated in general practice. These plans are tailor-made, using the feedback reports to guide their focus, and therefore be consistent with the needs of the practice. The program did provide additional support for developing and implementing improvement plans, which is important in applying elements of quality improvement. However, some participants experienced there was no possibility to implement their own priorities using improvement plans.

*‘What we disliked was that we were obliged to make a plan on the four most common chronic diseases, you have to do this, you have to do that. When you indicate you have other priorities for the improvement plans, you still have to implement a plan on topics they have made mandatory. That felt annoying sometimes.’ (respondent 13)*

#### Complexity

The first step in the practice accreditation program is collecting patient related data on four chronic diseases. Many participants experienced this as the most time-consuming and difficult step of the program. Furthermore, other elements of the program such as developing improvement plans and implementing these plans were experienced as a heavy burden as these are supplementary tasks in addition to daily practice.

*‘General practitioners prefer to do the things they enjoy, this (the practice accreditation program) takes a lot of extra time and workload.’(respondent 13)*

#### Design quality and packaging

The intervention consisted of a workbook, a supporting website and the obligation to contract a trained consultant to assist the practice through all steps of the program. Another component is the practice visit of an assessor to assess improvement plans and minimum standards. Practices in this study were in general not content with the supporting website which was found unclear and slow. Experiences with the assistance of the consultant varied. Furthermore there was a lack of consistency in assessment methods of assessors which caused confusion on how to interpret and execute the program.

#### Cost

All respondents expressed their dissatisfaction with the high costs of the intervention. Furthermore they questioned the benefits of the program, in particular in relation to the costs.

*‘We think it is a lot of work that has to be done, making improvement plans, evaluating the plans, it takes a disproportionate amount of time actually.’ (respondent 26)*

*‘I have to pay a considerable amount of money to do all kinds of things that I also would do without paying the money.’ (respondent 13)*

### Views on the outer setting

#### Patient needs and resources

In the practice accreditation program participants are obliged to conduct a patient satisfaction survey. Based on these outcomes several participants defined the aims of their improvement plans so that the needs of patients could be met.

*‘We used results from the patient survey to inspire us in choosing a topic for the improvement plans for this year. There were especially complaints regarding privacy.’ (respondent 26)*

However, participants perceived that patient care was not directly influenced by the accreditation program as the program had no direct influence on patient-caregiver interactions.

#### Cosmopolitanism

All practices in the study were affiliated with a chronic care group. In addition to the practice accreditation program, participants in the study mentioned participation in a chronic care group as a contextual factor that positively influenced the quality of the care they provided. Some of the practices were, as training practices, connected to an institute for vocational training of GPs. Peer review meetings for GPs working in training practices appeared to be a positive influence on their attitude towards quality improvement.

#### External policy and incentives

The most important extrinsic reasons to participate in the program were a financial stimulus for GP training practices and the requirement of insurance companies to demonstrate how quality is managed within the practice. Participation in research projects, nationally organized projects (on registration behavior) and participation in other certification programs all provided a positive influence on implementation of the program.

*‘One of our staff members was appointed for several hours a week to focus specifically on quality work. Not only for the practice accreditation program but also for the other certification program we participate in. These are preparations to ensure the whole team is involved in the process.’ (respondent 44)*

### Views on the inner setting

#### Structural characteristics

In small practice organizations, lines of communication were clear which is beneficial for the implementation of the practice accreditation program. However in solo-practices all tasks concerning the program had to be performed by one person. Furthermore, when a practice lost staff members due to illness or resignation, there was no priority for the program and it also implied the loss of knowledge about the program.

*‘Well, when you lose staff members because of resignation or illness, it causes major problems. First priority is to keep the practice running and then there is no time left to spend on tasks concerning the practice accreditation program.’ (respondent 46)*

The age of general practitioners was mentioned as a factor associated with the enthusiasm with which the program was accepted for implementation.

‘*I think it’s a generational thing. I have the feeling older GPs consider it more difficult to work according to the practice accreditation program than younger GPs.’ (respondent 15)*

#### Networks and communication

The practice accreditation program requires the involvement of the whole team. Therefore it is advised to organize structural team meetings to evaluate the progress of improvement plans. Participants experienced implementation of the program as more effective when indeed all members of the team were involved and processes were structurally evaluated in team meetings.

#### Culture

The majority of participants in this study had affinity with improving the quality of care they provided, prior to participating in the program, which benefits implementation. Furthermore participants mentioned that the motivation and education level of team members was of influence on the implementation of the program.

*‘I think we have team members with a critical attitude. All our assistants have a bachelors degree, which is uncommon.’ (respondent 34)*

#### Implementation climate

The degree of motivation regarding implementation of the program may be dependent on the function of the staff members. Some GP assistants experienced the program as a burden while practice nurses had no difficulties implementing the program. Furthermore, in some practices not all GPs believed the program is beneficial and were therefore less motivated to implement the program.

### Views on the characteristics of individuals

#### Knowledge and beliefs about the intervention

Participants started with the program while it provides support when improving quality of care in the practice:

*‘We wanted to be more conscious of the quality of care we provide and we wanted to reveal our blind spots. The most important reason to participate in the program was to improve the quality of care we provide.’ (respondent 24)*

#### Self-efficacy

The program provided tools to work systematically:

*‘I often started new things (new procedures) without completing them. The advantage of the accreditation program is that it forces me to complement the circles to implement new approaches in a constructive manner.’ (respondent 22)*

#### Individual stage of change

In the initial stages of the program participants required more assistance from the consultant than in later stages of the program. They then became more accustomed to working according to a quality cycle and the program was more integrated in daily practice.

#### Other personal attributes

Some participants were motivated to participate in the practice accreditation program because they were also employed in another function relating to quality of care.

### Views on the process of change

#### Planning

Most participants made no preparations before they volunteered to participate in the program. The practice accreditation program consists of various elements (Fig. 1). Practices in the program started with collecting patient related data to four chronic diseases (COPD, DM, CVRM, Asthma). Particularly this element was very time-consuming and led to barriers for some participants due to computer related problems.

*‘I’m no computer expert, I need help with that and I think that also applies to some of my colleagues’. (Respondent 38)*

Based on the data on four chronic diseases practices receive a feedback report with benchmarks which provides insight into their medical practice. This information was considered to be important however it had little influence on improvement plans. A possible reason is it is difficult to adequately reflect on the outcomes.

*‘It (the feedback report) shows the benefits of my efforts and indicates in what areas I should plan improvements. It is very difficult to reflect on the feedback reports sufficiently. I have to spend time to study it, to think about it and reflect on it. You should be able to discuss it with your team. The rush of daily practice leaves no time for this and that is very unfortunate.’ (Respondent 44)*

However, some participants considered the feedback report of minor importance.

*‘No, we do not look into it that much. This is our practice and we manage it in our own way.’ (Respondent 29)*

Another element of the practice accreditation program was the formulation of improvement plans. These plans were in general drafted and implemented by all team members. The practice consultant provided useful feedback on the plans in the first year of the program. However, the subject of improvement plans can be determined to a certain extent only.

‘*I have to come up with three new plans for this year. You have to be careful you don’t make up things only because the auditor is coming.’ (Respondent 45)*

Visitation of an assessor is the next element of the practice accreditation program. During this visit the assessor audits the practice. Results of the audit seem to depend on which assessor visits the practice.

*‘I have noticed over time that the assessors all have different backgrounds. They assess the practice in a non-similar manner. The things that are important differ for various assessors.’ (Respondent 34)*

#### Engaging

Some of the partners of the GPs were team member of the practice team. This appeared to be a highly stimulating factor in implementation of the program.

*‘As the manager I have the time to perform accreditation-related tasks. So I took the initiative, otherwise it would not have been a success. We talk about it over dinner, so to speak, so the reflection process is already started. And then at one point I nag that he really has to write those plans, and then he picks up the voice recorder and begins.’ (Respondent 29)*

When starting with the program some participants expanded responsibilities of other team members for the purpose of guiding the implementation of the program.

*‘One of our assistants had just finished a management training, that was our benefit. We appointed her as coordinator of the practice accreditation program.’ (Respondent 40)*

#### Executing

Every year the practice is audited by an auditor who assesses the objectives of implemented improvement plans and approves new improvement plans. This annual visit is for most participants an important motivator for continuous quality improvement and to keep implementation of improvement plans on the practice agenda.

#### Reflecting and evaluating

Quantitative feedback about the progress of implementation of the program was provided by feedback reports at the start of the program. It is required for practices to define their improvement plans with a measurable goal. After a year they have to provide evidence that goals have been achieved. Furthermore team meetings were regularly held, as required by the practice accreditation program, to monitor progress of implementation.

As a result of participation in the program, team members were more motivated in performing their work and their responsibility increased as a consequence of participation in the program. Overall a better team spirit emerged.

*‘Very often issues are not mentioned because it is difficult to give one another feedback. Now we succeeded in establishing a safe work environment where we can provide each other feedback in a constructive manner.’ (respondent 40)*

## Discussion

The aim of this study was to identify determinants of impact of the practice accreditation program, building on the experiences of primary care professionals who had participated in a practice accreditation program in primary care. The presence of a team member who has the specific responsibility for the program, appeared to be a stimulating factor. The practice accreditation program had positive effects on team climate and caused more sense of responsibility for quality of care among all team members. Health care professionals perceived no direct influence by the accreditation program on patient care. Audit and feedback is a crucial element of the accreditation program, however choices for improvement plans were rarely based on feedback reports. Receiving a certificate for completing the accreditation program seemed to have little value to participants.

As shown in a Cochrane review [15] audit and feedback leads to variable and overall modest improvements in professional practice. The effectiveness seems to depend on baseline performance and on how feedback is exactly provided. Knowledge gaps remain regarding when audit and feedback will work best and why [16]. Feedback is more effective when accompanied by both explicit goals and an action plan. However, results in this study show that feedback is not necessarily used when making improvement plans, because practices have ideas in advance on what to improve regardless of the outcomes of feedback. Furthermore, external factors, such as participation in chronic care groups, appear to have more important impact on the implementation of new or improved procedures in the practice than audit and feedback. As shown in this study, participants experienced that patient care was not directly influenced by the accreditation program, it is therefore recommended that improvement plans should be focussed more on improvement of outcome measures.

In this study staff responsibility for quality was identified as an important implementation facilitator, which was also demonstrated in a previous study [17]. Similar to previous studies in hospital-settings [18], this study in primary care demonstrates contrasting professional attitudes towards accreditation programs; a possible explanation for this may be the age of the professional [19]. The program results in better organizational performance and it provides a guide to external stakeholders illustrating how quality is managed within the practice. However critical perspectives are that the program is bureaucratic, time consuming and adds little value to patient care because of its focus on administrative processes. Furthermore there is a perceived lack of consistency among assessors.

As a response to this and other evaluations, the Netherlands Institute for Accreditation in Healthcare has adjusted the practice accreditation program to make implementation more feasible and flexible. Data collection has been spread over different years, improvement plans can be documented in a more flexible way, and the use of external advisors is optional. New evaluation is required to examine the impact of these changes on feasibility and outcomes.

### Strengths and limitations of the study

The strength of this study lies in the qualitative approach which gives us more information on the working elements of the practice accreditation program. The CFIR framework was used to organize the data in this study. All domains described in the CFIR were represented in the results. As the CFIR framework was only used in the last stage of coding, the risk of overlooking material that does not fit in the constructs, was small. General practices in this study all voluntarily applied for the practice accreditation program and subsequently they voluntarily participated in this study. This could imply that participants included in this study had a more than average affinity with quality of care and were motivated to change. Therefore it is recommended to conduct further research in the late majority population. In this study only the quality coordinators of the practices were approached for participation. A focus group study with all team members of the practice could have resulted in additional outcomes to provide more understanding of mechanisms of action regarding implementation of the program. Furthermore, the data collection method we choose might have been inadequate as with face-to-face interviews more in-depth and nuanced data can be collected.

This study contributes to the body of knowledge on determinants of impact of a practice accreditation program implemented in general practice. Determinants perceived of importance in this study have to be further explored in future research. However, the results of this study provide feasible, ready to use suggestions, and therefore can be relevant for general practice teams, practice managers and policymakers. An example of such a ready to use suggestion is: designate one responsible team member, to facilitate the implementation of a practice accreditation program.

Consistent with results from this study, previous research has shown that accreditation results in improved teamwork, improved access to care, increased awareness of patient safety, improved practice systems and care processes and improved quality of care [19]. Nevertheless, not all planned elements of accreditation appeared to contribute to its outcomes, so there may be room for improving efficiency of the program. As shown in this study, elements that were perceived to facilitate implementation of the program were; designating one person responsible for the program, ensuring clear lines of communication within the whole practice team, and having affinity with or stimulate enthusiasm for improving quality of care. Furthermore, contextual factors such as participation in a care group and being connected to the GP educational institute were important for practice change. The importance of the elucidation of contextual factors has been shown in previous research. Reporting contextual information is a way to provide information needed to foster health care systems [20], and it is therefore recommended to explore contextual information in future accreditation research.

Across the world, practice accreditation is an established strategy for assessing and improving healthcare practices. Nevertheless, there remains a need for better insight into the factors and processes related to its impact in order to optimize existing accreditation programs.

## Additional file

Additional file 1: ᅟ
